# Multiple ectopic parathyroid adenomas

**DOI:** 10.1590/S1516-31802004000100008

**Published:** 2004-01-08

**Authors:** Dedivitis Rogério Aparecido, Guimarães André Vicente, Gustavo Bastos de Goes Pontes

**Keywords:** Parathyroid glands, Hyperparathyroidism, Parathyroidectomy, Parathyroid neoplasms, Glândulas paratireóides, Hiperparatireoidismo, Paratireoidectomia, Neoplasias das paratireóides

## Abstract

**CONTEXT:**

Primary hyperparathyroidism is the most common cause of hypercalcemia in unselected patients. The ectopic gland locations should be known for appropriate surgical exploration and for avoiding subsequent re-exploration that would represent higher morbidity. Multiple ectopic glands are rare and present a particular challenge in parathyroid surgery.

**CASE REPORT:**

A 65-year-old female presented with nephrolithiasis. Her serum total calcium was found to be elevated. The diagnosis of primary hyperparathyroidism was confirmed by the elevated serum intact parathyroid hormone levels. Ultrasound was only successful in localizing one adenoma in the lower right gland. Technetium sestamibi scanning correctly localized the same adenoma and showed another contralateral image, lateral to the thyroid cartilage. Fiber optic laryngoscopy showed an extrinsic mass pushing against the lateral and posterior walls of the left pyriform sinus. Resonance imaging revealed a soft tissue mass.

**RESULTS:**

The patient underwent bilateral neck exploration. Histopathological examination confirmed the diagnosis of parathyroid double adenomas. The late-stage postoperative checkups were normal.

**DISCUSSION:**

Routine bilateral neck surgery should be performed as a rule. We use ultrasound and technetium sestamibi scanning as a routine for preoperative localization studies. It is helpful to have an experienced surgeon for the localization.

## RESUMO

**CONTEXTO:**

Hiperparatireoidismo primário é a causa mais comum de hipercalcemia em indivíduos examinados aleatoriamente. A localização das glândulas ectópicas deve ser conhecida para uma exploração cirúrgica apropriada e para evitar subseqüente re-exploração, o que representa um índice de morbidade maior. Múltiplas glândulas ectópicas são raras e apresentam um desafio particular à cirurgia das paratireóides.

**RELATO DE CASO:**

Paciente do gênero feminino de 65 anos apresentou nefrolitíase. A dosagem de cálcio sérico estava elevada. O diagnóstico de hiperparatireoidismo primário foi confirmado pela elevação do paratormônio intacto sérico. O ultra-som só obteve sucesso em localizar um adenoma na glândula inferior direita. Cintilografia por tecnécio-Sestamibi localizou corretamente o mesmo adenoma e outra imagem contralateral, lateralmente à cartilagem tireóidea. Laringofibroscopia mostrou uma massa extrínseca empurrando as paredes lateral e posterior do recesso piriforme esquerdo. Imagens de ressonância revelaram uma massa de tecido frouxo.

**RESULTADO:**

Ela foi submetida a uma exploração de pescoço bilateral. O exame histopatológico confirmou o diagnóstico de adenoma duplo de paratireóide. O controle pósoperatório foi normal.

**DISCUSSÃO:**

Exploração cervical bilateral rotineira deve ser executada. Nós usamos ultrasom e tecnécio-Sestamibi como uma rotina de estudos pré-operatório de localização. A experiência do cirurgião é importante na localização.

## INTRODUCTION

Primary hyperparathyroidism is the most common cause of hypercalcemia in unselected patients. The most common cause is a single adenoma (80-85%), while multiple gland hyperplasia occurs in 12-15%, double adenoma in 2-3% and carcinoma in about 1%.^1^ Ectopic adenomas, unrecognized hyperplastic glands, and supernumerary glands are considered to be the causes of failure after the initial cervical exploration. Ectopic multiple glands are rare and present a particular challenge in parathyroid surgery. The frequency of multiple parathyroid glands in ectopic locations in the same patient is not well described in the literature, thus suggesting that they are uncommon.^2^ We report on a patient with primary hyperparathyroidism with an unusual presentation of ectopic and double adenoma.

## CASE REPORT

A 65-year-old previously healthy woman presented with an episode of unilateral renal colic associated with hematuria. She was found to have nephrolithiasis and was clinically treated. The physical examination was unremarkable. Her serum total calcium was found to be elevated, confirmed as 12 mg/dl and 11.7 mg/dl. Other results showed serum phosphate of 1.3 mg/dl and alkaline phosphatase of 550 U/l. The diagnosis of primary hyperparathyroidism was confirmed by elevated serum intact parathyroid hormone levels of 95 pg/ml.

**Figure 1:**
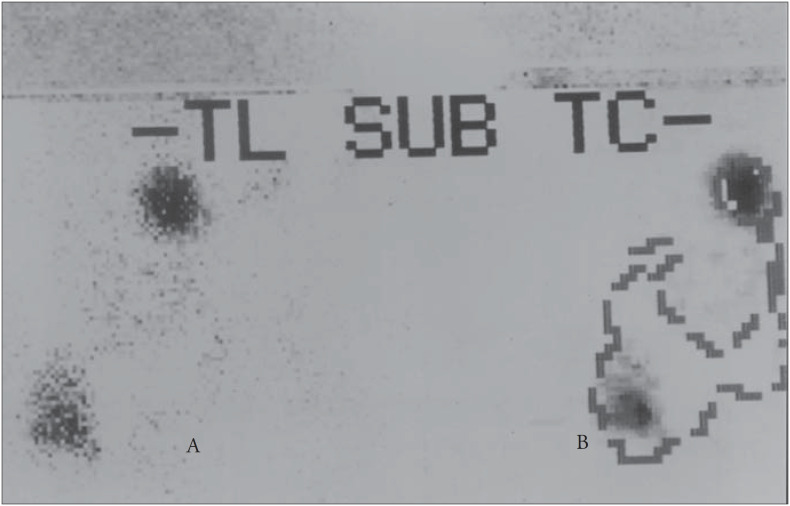
(A) Technetium sestamibi scan, correctly localizing both adenomas; (B) Thyroid silhouette superimposed over the representation of the adenomas.

**Figure 2:**
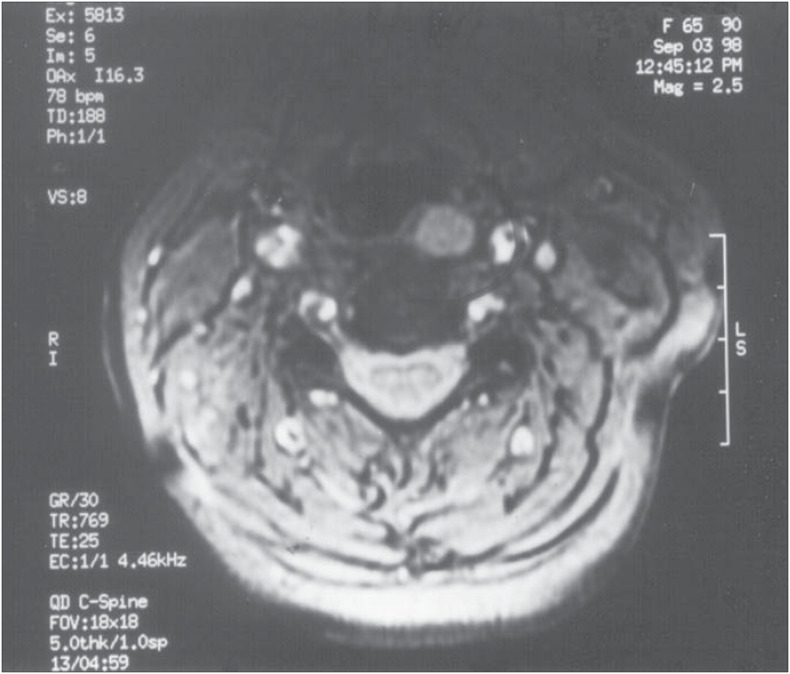
Resonance image showing a soft tissue mass pushing against the lateral and posterior walls of the left pyriform sinus.

Ultrasound was successful in localizing one adenoma in the lower right gland. Technetium sestamibi scanning correctly localized the same adenoma and showed another contralateral image, lateral to the thyroid cartilage (Figure 1). Fiber optic laryngoscopy showed an extrinsic mass pushing against the lateral and posterior walls of the left pyriform sinus, measuring about 1 cm. As this presentation was unusual, the patient underwent resonance imaging that revealed a soft tissue mass in that location (Figure 2).

The patient was admitted to hospital for a surgical procedure. The upper right and left glands were identified in their normal anatomical positions and their sizes were also normal. The adenomas were located at a topical lower right position and just below the left pyriform sinus. No parathyroid gland was identified in the anatomical position of the lower left gland. Histopathological examination confirmed the diagnosis of parathyroid adenomas for both lesions that were removed.

The postoperative serum calcium levels normalized within 24-48 hours and the patient presented symptomatic hypocalcemia due to the "hungry bone" syndrome, which was controlled clinically. The patient was discharged with normal serum calcium and normal serum phosphate on the third postoperative day. She started on oral calcium carbonate medication, which was stopped in the second postoperative week. The late-stage post-operative checkups were normal.

## DISCUSSION

Multiple and/or ectopic adenomas are rare causes of primary hyperparathyroidism. The role of preoperative localization studies remains controversial. Previous positive studies increase surgical confidence and reduce operating time. However, the routine localization of ectopic adenomas is unreliable via ultrasound. In addition, both isotope scans and ultrasound produce false negative and false positive results.^1^ Sestamibi scans have been least accurate in patients with multiple abnormal glands.^3^

Abnormal parathyroid glands can be identified during initial surgical exploration by experienced surgeons in about 90-95% of cases. Bilateral neck exploration should be performed in every case, regardless of the preoperative localization methods. A careful search should also be conducted at the most frequent ectopic sites^4^ . Fourteen percent of adenomas are found at ectopic locations. An ectopic upper parathyroid gland is most commonly found at retroesophageal sites. An ectopic lower gland is more likely to be found in the anterior mediastinum, in association with the thymus gland.^1^
